# Spontaneous Pneumomediastinum Associated With SARS-CoV-2: Infrequent Complication of the Novel Disease

**DOI:** 10.7759/cureus.9189

**Published:** 2020-07-14

**Authors:** Urszula Wegner, Gerardo Jeffery, Octavio Abrajan, Italo Sampablo, Chitrangada Singh

**Affiliations:** 1 Radiology, King George Hospital, Barking, Havering and Redbridge (BHR) University Hospitals, London, GBR; 2 Radiology, Hospiten Cancun, Cancun, MEX; 3 Internal Medicine, Hospiten Cancun, Cancun, MEX; 4 Radiology, Norfolk and Norwich University Hospital, Norwich, GBR

**Keywords:** pneumomediastinum, coronavirus, pneumonia, sars-cov-2, pandemic

## Abstract

A 44-year-old male with no previous medical history or comorbidities presented with significantly increasing shortness of breath, myalgia, nausea, and fatigue. He had no diagnosed medical conditions and enjoyed good health prior to the episode of acute respiratory infection. There was no history of smoking, emphysema, or chronic lung diseases. CT revealed bilateral ground-glass opacities in predominantly peripheral distribution. Based on imaging spectrum and global pandemic of the novel coronavirus, typical SARS-CoV-2 (severe acute respiratory syndrome coronavirus 2) infection was suspected. Viral load was confirmed with biochemical data and laboratory results. Interestingly, despite intensive treatment, the patient developed sudden complications during the second week of his hospitalization. The symptoms started to resolve on pharmacological treatment and supplemental noninvasive oxygen supply over the next weeks.

We illustrate and discuss the case of spontaneous pneumomediastinum as an uncommon manifestation of novel SARS-CoV2 chest infection. Even though our patient did not develop acute respiratory distress syndrome or further complications, the presented case highlights the importance of basic radiological monitoring of the disease in order to ensure prompt diagnosis of complications and appropriate subsequent management.

## Introduction

The World Health Organization (WHO) announced a fast-evolving and an alarming outbreak of the coronavirus disease 2019 (COVID-19) pandemic when confirmed cases approached 200,000 in 160 countries across the globe [1].

The characteristic symptoms of the novel virus mostly include fever, cough, and loss of smell and taste [2,3]. However, clinical manifestations may be different in various age groups [1,2]. Classic imaging features include bilateral, peripheral predominant ground-glass opacities or peripheral consolidations often with multilobar involvement [3].

The presented case of spontaneous pneumomediastinum is a relatively rare complication of a more severe course and advanced stage of the novel SARS-CoV-2 (severe acute respiratory syndrome coronavirus 2) pulmonary infection, especially in the absence of emphysema or any underlying pulmonary disease. It can occur in affected patients regardless of their age, prior health conditions, or coexisting respiratory diseases [3-5]. This can be unilateral and usually may develop in the setting of a progressive main infectious disease with pneumonic infiltrates typical of COVID-19 related imaging spectrum. The condition usually manifests as progressive shortness of breath with subsequent hypoxia and can be easily diagnosed on CT or even on a classical radiograph.

Awareness of the possible course of COVID-19 pneumonia and potential complications is essential for physicians to contribute to safe and effective management, is the key to faster recovery, and plays a crucial role with regard to the therapeutic outcome of severely ill patients. Elderly patients are at higher risk of severe course of pneumonia and complications in advanced stages of novel disease [2]. However, the exact incidence and data regarding spontaneous pneumomediastinum in the affected general population are not available.

This emphasizes the significance of a more attentive approach and usefulness of follow-up CT scans to ensure that there is no delay in the diagnosis of associated complications during the course of acute COVID-19 pneumonia. Classical radiographs are of less diagnostic value as findings can be missed in the early phases of disease, whereas CT can demonstrate changes even prior to clinical onset of infection [2,3].

## Case presentation

A 44-year-old male with no previous medical history presented with fever, dry cough, and worsening shortness of breath. There were no risk factors, previous complaints, asthma, or any chronic diseases. He never smoked, had no surgical history, and was healthy prior to the current acute respiratory episode.

The patient was admitted to the emergency department for further evaluation. His oxygen saturation was 91% on admission and the temperature was 39 Celsius degrees. Standard laboratory tests revealed that C-reactive protein (CRP) was elevated to 14.1 mg/dL, and leukocytes were slightly increased up to 13.53 x 10^9^/L. The patient’s condition started to deteriorate on admission, and non-invasive ventilation through a face mask was delivered to improve gas exchange. This helped to improve dyspnea, and he did not require endotracheal airway or invasive ventilation. High-resolution CT (HRCT) scan was subsequently requested, which revealed bilateral peripheral predominant ground-glass opacities. No overt features of chronic disease, fibrosis, or bullous emphysema were demonstrated. Acute clinical presentation in an otherwise healthy person in the background of the global SARS-CoV-2 pandemic was suggestive of the novel coronavirus-associated respiratory illness. This correlated well with the real-time polymerase chain reaction test (RT-PCR) performed.

**Figure 1:**
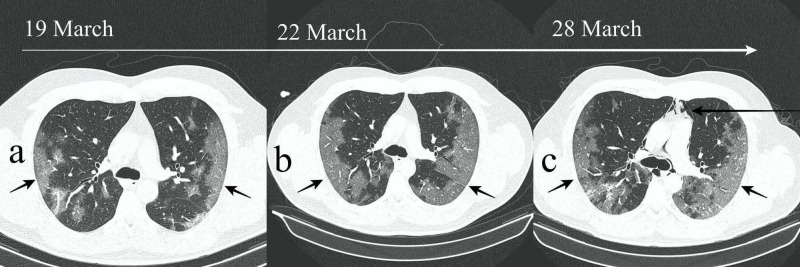
(a-c) Axial HRCT images demonstrating progressive and peripheral predominant ground-glass opacities consistent with COVID-19 (short arrows). Spontaneous pneumomediastinum developed during the second week (long arrow). HRCT, high-resolution CT

Imaging findings were reported as classic COVID due to the visualized characteristic bilateral peripheral ground-glass consolidations (Figures 1a-1c, short arrows). Pulmonary opacities were very extensive, thus the severe score was assigned as per the Chest CT Severity Score, which is used for assessing parenchymal involvement. The disease rapidly progressed over the next 10 days (Figures 1a-1c, short arrows). There was no pericardial or overt pleural effusion.

Despite initial shortness of breath and severe CT score, invasive ventilation was not required as supplemental oxygen was provided through oronasal mask, which significantly improved patient’s comfort and reduced respiratory effort. Our patient was given intensive pharmacological treatment with 300 mg atazanavir every 24 hours for seven days, 500 mg clarithromycin every 12 hours for seven days, and 400 mg hydroxychloroquine every 12 hours for five days.

**Figure 2:**
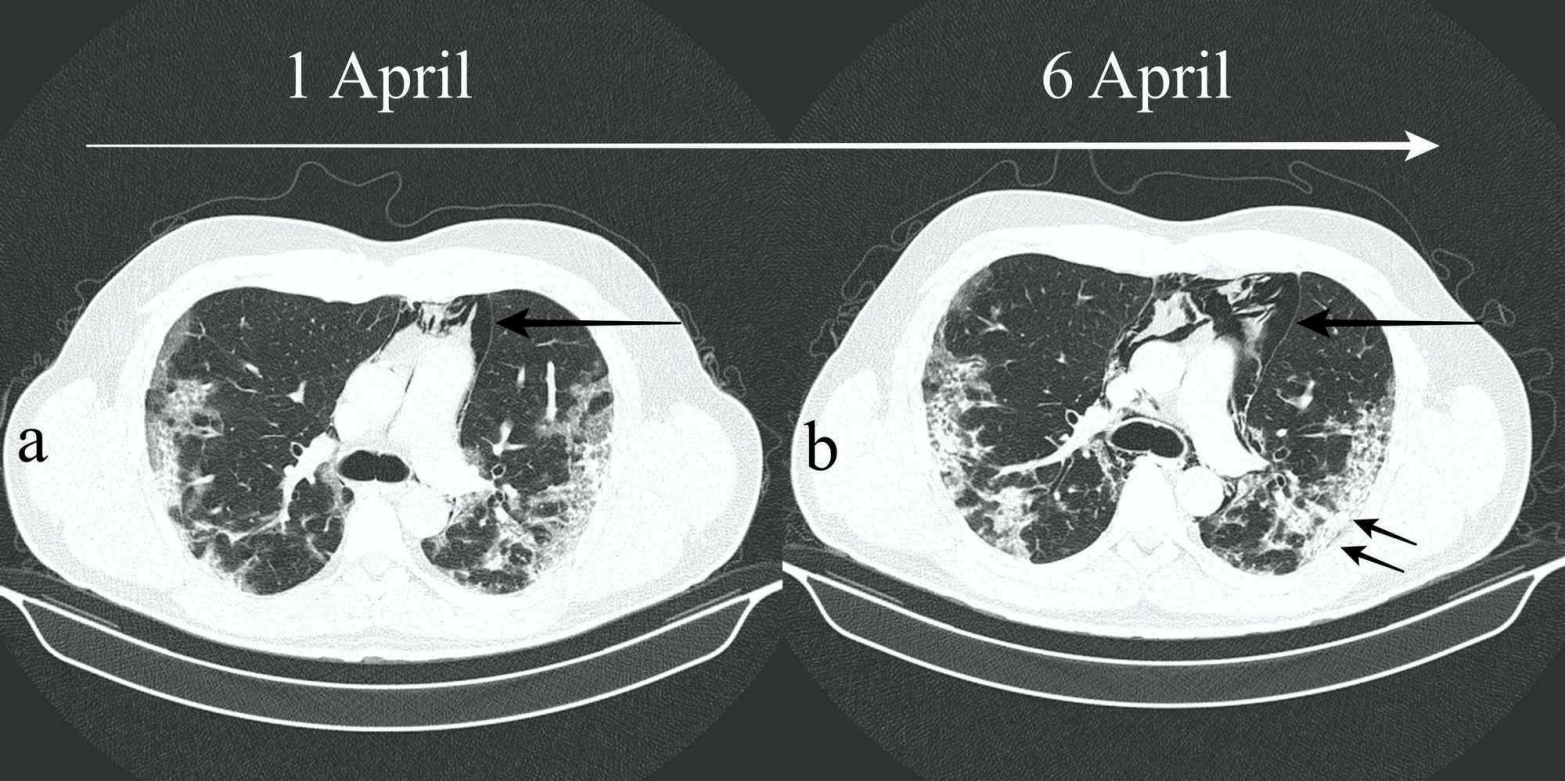
(a,b) Axial HRCT images showing progressive pneumomediastinum during the course of the novel disease (long arrows). In addition, previously noted ground-glass opacities started to progress into denser consolidations (short arrows). HRCT, high-resolution CT

However, there was a sudden unexpected onset of more intensive acute respiratory symptoms during the second week of hospitalization; thus, serial CT scans were requested to closely monitor the evolution of disease. The third scan revealed spontaneous and progressive pneumomediastinum (Figures 1c, 2a, 2b, long arrows).

Bilateral ground-glass opacities became denser in comparison to initial examination (Figures 2a, 2b, short arrows). Spontaneous pneumomediastinum was an unexpected complication during the course of severe SARS-CoV2 related pneumonia in an otherwise healthy patient without any risk factors. Surprisingly, our patient had no background of asthma, emphysema, or any signs of chronic lung disease. Despite exacerbation of hypoxia, no neurological or cardiovascular symptoms were observed. There was no septic shock, renal injury, or multiple organ dysfunction syndrome. Arterial blood pressure was within normal limits. No other immediate complications were observed.

**Figure 3:**
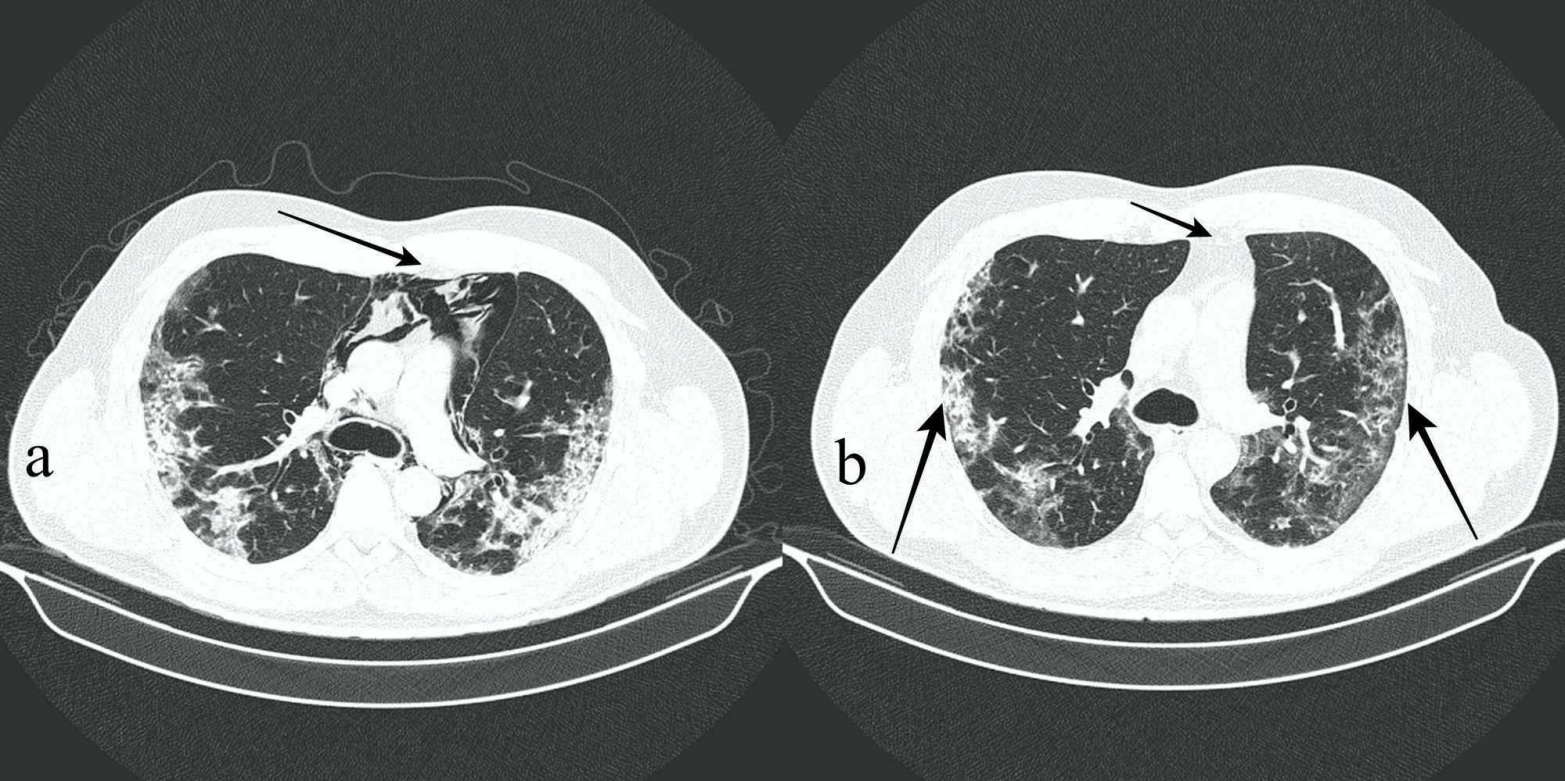
(a,b) Axial HRCT images demonstrating full resolution of spontaneous pneumomediastinum after one month since initial acute symptoms (b, short arrow). However, residual mixed attenuation bilateral and peripheral predominant opacities were still present (b, long arrows). See significant volume pneumomediastinum in the third week of disease for comparison (a, long arrow). HRCT, high-resolution CT

**Figure 4:**
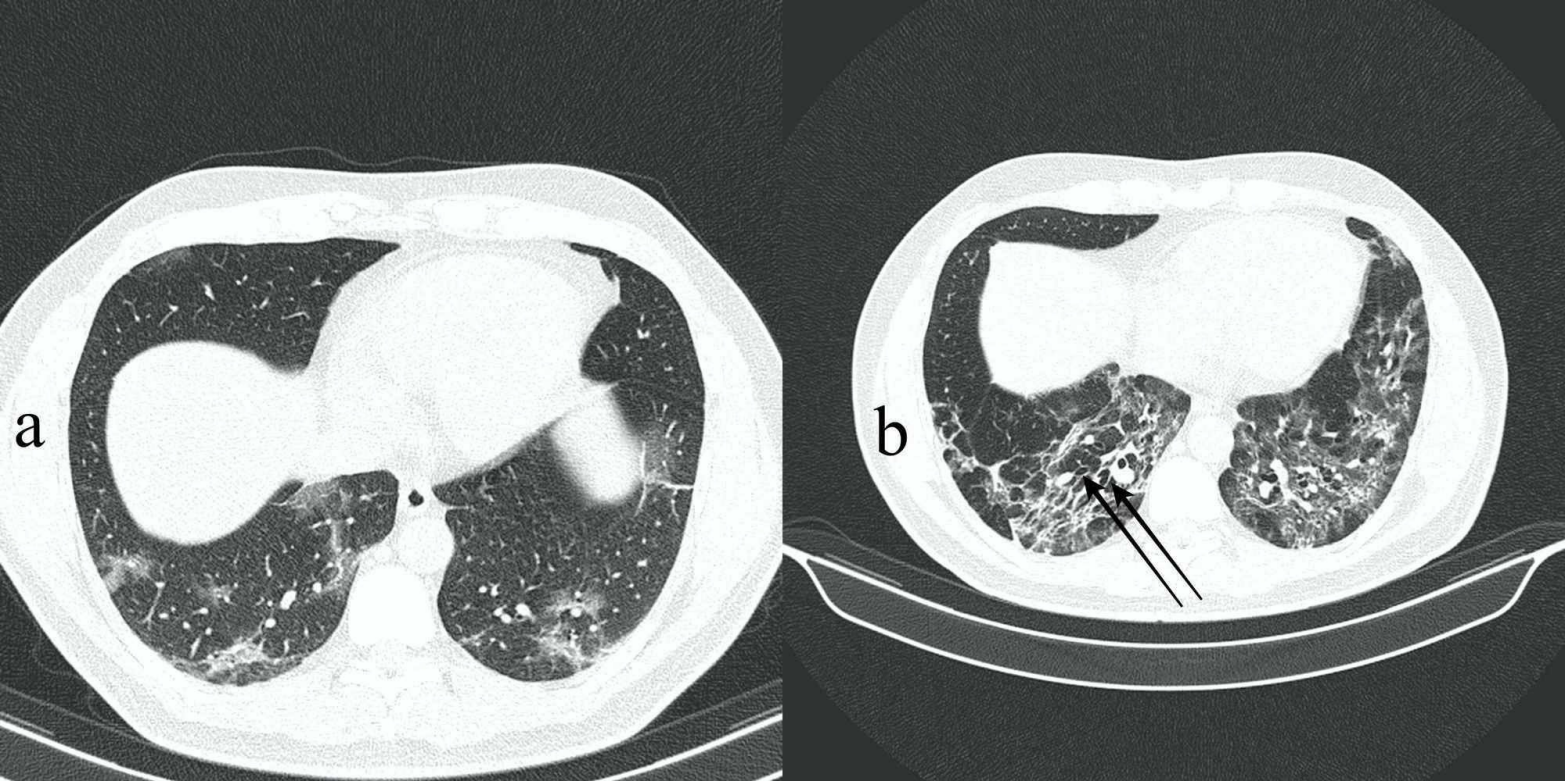
(a,b) Axial HRCT images showing new bronchiectasis and fibrotic changes as a sequel to severe respiratory disease (b, arrows). See initial baseline scan 35 days earlier without bronchiectasis for comparison (a). HRCT, high-resolution CT

Respiratory symptoms started to improve slowly and pneumomediastinum fully resolved after four weeks (Figures 3a, 3b, arrows). Interestingly, inflammatory markers normalized, and CRP decreased from 14.1 to 0.8 mg/dL after 16 days. Gradual and slow but satisfactory clinical and radiological recovery was observed. A final HRCT scan was requested to ensure further resolution and improvement regarding previously observed residual mixed attenuation peripheral opacities (Figure 3b, long arrows). This was performed on the 35th day of hospitalization and revealed new bronchiectasis and fibrotic changes as a sequel to severe COVID-19 pneumonia (Figure 4). The patient was discharged after 35 days of hospitalization.

Since long-term effects of the novel disease remain unknown, our patient was informed of potential possibility and risk of subsequent permanent injury to the lung parenchyma and was requested to come to the hospital if any new respiratory symptoms occur in the future.

## Discussion

Confirmed infections of novel SARS-CoV-2 were initially reported in December 2019 in Wuhan in China, and since then the highly contagious viral disease started to spread rapidly to almost all other parts of the world [1,3]. The WHO declared the global pandemic when confirmed cases reached almost 200,000 across 160 countries, as the situation in the setting of novel COVID-19 was dynamically evolving and challenging health systems worldwide [1]. The most common clinical key indicators of the virus include cough, fever, and other well-established symptoms such as anosmia and ageusia [2,3]. Typical imaging appearances are of bilateral ground-glass opacities in peripheral predominant distribution [2]. Ground-glass opacities can transform into consolidations in advanced ongoing disease [2,3]. Mediastinal lymphadenopathy, pleural effusions, or complications such as pneumothorax and pneumomediastinum are less typical and may appear usually during the course of progressive pulmonary infection [2,3].

Significant pneumomediastinum in the presented case appeared in the end of the second week and was associated with progressive multiple consolidations. In addition, there were no other acute complications that would require invasive ventilation. A similar case was reported by Zhou et al. in March 2020. Interestingly, spontaneous pneumomediastinum also developed in the second week of severe pneumonia [4]. However, our patient had no associated cardiovascular symptoms as opposed to the previously reported case. The timeframe with regard to clinical recovery and complete resolution of pneumomediastinum was similar in both cases, approximately one month (Table 1). Moreover, both patients were young and healthy and developed pulmonary fibrosis as a sequel to severe respiratory infection [4].

**Table 1:** Comparison with regard to patients with COVID-19 who developed complications during progressive and severe course of respiratory infection.

	Age and sex	Comorbidities	Complications and time of development	Hospitalization
Zhou et al. [4]	A 38-year-old male	None	Pneumomediastinum 11th day	30 days
Sun et al. [6]	A 38-year-old male	None	Pneumomediastinum 26th day, pneumothorax 34th day	34 days
Presented case	A 44-year-old male	None	Pneumomediastinum 9th day	35 days

Spontaneous pneumomediastinum is a rare and self-limiting condition, which manifests as pleuritic pain and shortness of breath. Frequently, patients who present with such complications have either a history of asthma, chronic obstructive pulmonary disease, or infections, or inhale cocaine. This condition usually gradually resolves within a few weeks, and no invasive interventions are required [5]. Since there are limited literature resources and reports on potential complications associated with COVID-19 pneumonia, overall incidence is not yet fully well-established. Apart from spontaneous pneumothorax, pneumomediastinum or subcutaneous emphysema can infrequently be observed [6].

An interesting case of complex course of novel respiratory disease with spontaneous left-sided pneumothorax has recently been reported and illustrated by Sun et al. Their patient also had no previous respiratory conditions or risk factors. However, the latter was associated with a more severe and progressive disease, and pneumomediastinum was accompanied by significant pneumothorax (Table 1) [6].

Interestingly, our patient demonstrated both radiological and clinical deterioration and developed unexpected complications related to pneumomediastinum but without pneumothorax. Moreover, all the three discussed patients were relatively young males with no history of comorbidities, who developed serious complications and had similar timeframe with regard to hospitalization (Table 1).

Importantly, COVID-19 related infection can be associated with a number of other complications including neurologic manifestations, neuropathies, myopathy, seizures, and even stroke [7]. However, the majority of dramatic neurologic complications usually appeared in patients with a more severe course of disease or with other predisposing comorbidities [7]. It is important to highlight that long-term characteristics and future implications of the novel viral disease remain unknown yet. SARS-CoV-2 can easily spread from human to human, and, moreover, there is preliminary evidence that the virus can linger in the air for hours in the form of aerosols predisposing to airborne transmission, which could explain the rapid overwhelming explosion of the pandemic across the world [8,9]. In addition, there is no established treatment or antiviral medication that could definitely improve patient outcome and decrease mortality of suffering patients [10,11]. Thus, timely management, identification of any coexisting complications, risks related to the novel disease, and its possible clinical manifestations are of high significance.

## Conclusions

The presented interesting course of disease shows that the novel COVID-19 respiratory infection may be associated with complex and various clinical manifestations including development of pneumomediastinum or subcutaneous emphysema in otherwise healthy people who enjoyed good health prior to contracting the coronavirus. Familiarity with demonstrated imaging spectrum and possible complications is crucial to ensure appropriate surveillance and timely treatment.

It is important to highlight that both spontaneous pneumothorax and spontaneous pneumomediastinum may occur in the absence of emphysema, bullae, or chronic lung disease, and indicate a more severe and progressive respiratory infection. The described review of cases demonstrates that young and otherwise healthy male patients can develop serious complications and require a relatively long time of hospitalization of approximately one month since the onset of acute symptoms. This reinstates the value of radiological monitoring with CT in correlation with clinical presentation and serial imaging to rule out complications on time and to ensure a favorable evolution of the acute viral disease.

Since there is no vaccination yet or definite guidelines regarding the treatment of the novel SARS-CoV-2 infection, careful approach and knowledge of various potential clinical scenarios are of the greatest possible importance. Prompt reaction to and treatment of coexisting arising complications are crucial to survival and positive therapeutic outcome of the affected patients and may be helpful in decreasing the fatality rate.
